# Oral Management of a Haematopoietic Stem Cell Transplant Recipient with Chédiak–Higashi Syndrome

**DOI:** 10.1155/2021/9918199

**Published:** 2021-09-20

**Authors:** Kasumi Shimizu, Miho Hayashi, Nozomi Ito, Kokoro Hamada, Gaku Koizumi, Kazuto Kurohara, Naoya Arai

**Affiliations:** Department of Oral and Maxillofacial Surgery, Department of Clinical Sciences, Medical Life Science, Mie University Graduate School of Medicine, Tsu, Mie 514-8507, Japan

## Abstract

Chédiak–Higashi syndrome (CHS), a rare autosomal recessive disorder associated with leukocyte dysfunction, is characterised by partial skin and hair albinism, immunodeficiency, and abnormal bleeding. Furthermore, it may be associated with cognitive and neurological impairments. The long-term prognosis of patients is generally poor, and haematopoietic stem cell transplantation is a radical immunodeficiency treatment. Here, we report a case of successful oral management of an 18-year-old woman with CHS accompanied by aggressive periodontitis who underwent haematopoietic stem cell transplantation.

## 1. Introduction

Chédiak–Higashi syndrome (CHS) is a rare congenital recessive disorder caused by mutations in the lysosomal trafficking regulator gene (*LYST*) [1]. A murine model of CHS suggested that *LYST* encodes a cytoplasmic protein and is involved in granule transport and fusion; however, the exact cellular function of this protein is unclear. Histologically, mutations of *LYST* result in large intracellular granules that are characteristic of CHS. The encapsulation of giant melanosomes and large amounts of lysosomes can be observed in melanocytes and leukocytes, respectively, and are associated with hypopigmentation and impaired cellular immunity. Defective organelle transport also markedly reduces the number of dense granules present in platelets [2].

Only approximately 500 cases of CHS have been reported worldwide. CHS has multiple clinical features, including hypopigmentation of the skin and hair, reduced platelet and leukocyte counts, abnormal organelles in circulating granulated cells, and neurological dysfunction. Patients often exhibit a severe phenotype and die in the first decade of life; however, in approximately 10%–15% of patients, the disease has a relatively milder clinical course.

Even patients with comparatively mild CHS reportedly experience severe periodontitis due to immunodeficiency. Thumbigere Math et al. [3] reported that *LYST* mutations in patients with CHS can affect the expression and function of Toll-like receptor 2 and Toll-like receptor 4, leading to dysregulation of immune reactions and affecting the status of periodontitis.

Therefore, oral management is of utmost importance in patients undergoing haematopoietic stem cell transplantation. However, few reports have discussed oral management in patients with CHS undergoing haematopoietic stem cell transplantation.

In this study, we describe the oral management for a patient with CHS who had severe periodontitis before undergoing haematopoietic stem cell transplantation.

## 2. Case Presentation

**Figure 1:**
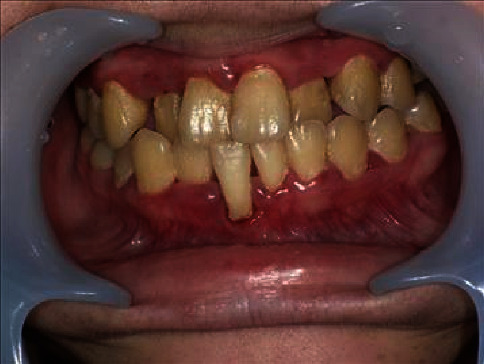
Intraoral view at the initial visit. Gingival swelling and redness were present throughout the oral cavity, and gingival retraction of the right lower central incisor was evident.

An 18-year-old woman was referred to our department in a wheelchair for oral management before undergoing bone marrow transplant. She was diagnosed with atypical CHS 3 years prior by LYST RNA analysis and based on her clinical course. Because of repeated infections, she was scheduled to undergo haematopoietic stem cell transplantation in our paediatrics department after the completion of treatment for her limb pain, which is one of the neurological symptoms of CHS. At her first visit, the general findings included hyperpigmentation on the facial skin, which is often seen in Asian CHS. Her intraoral examination revealed extensive gingival swelling and redness and gingival retraction in the right lower central incisor (Figure 1). The gingival pocket depth was 5–6 mm, the degree of movement was grade 1, and the O'Leary plaque control record (PCR) value was 80% (Table 1).

**Figure 2:**
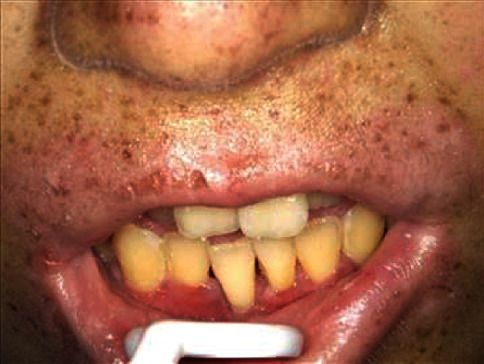
Oral condition 2 weeks after stem cell transplantation. Oral mucositis improved slightly. Hyperpigmentation was noted around the lips, but this was also present at the first visit.

**Figure 3:**
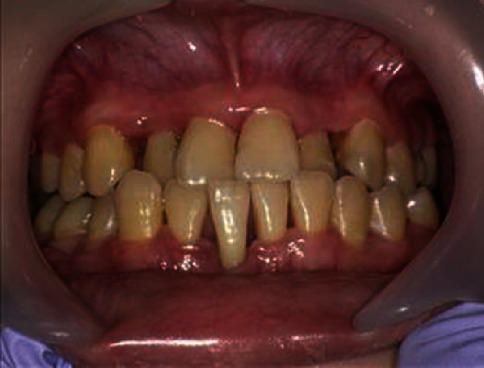
Oral condition 7 weeks after stem cell transplantation. Mild gingival swelling was evident, and the redness had improved. Bleeding was absent during scaling or brushing.

We initiated professional oral care and self-administered oral care education 7 weeks before transplantation (Table 2). Her intelligence quotient was 75, and she feared using the ultrasonic scaler, so we made an effort to eliminate her fear. We performed professional mechanical tooth cleaning and cleaned the periodontal pockets. The O'Leary PCR value was very high on her first visit. However, it decreased following the educational sessions, and it was 58% at the final session before bone marrow transplantation. Approximately 2 weeks before transplantation, she entered the cleaning room. Etoposide, busulfan, and cyclophosphamide were administered, and grade 1 mucositis was observed as a side effect on the day before transplantation. The Common Terminology Criteria for Adverse Events version 4.0 was used to evaluate mucositis. From the day after transplantation, redness was observed on her oral mucosa as a side effect of methotrexate, and she experienced pain that rendered brushing impossible (Table 3). A gargle containing lidocaine (Xylocaine®) was effective. The use of Episil® oral liquid was also attempted, but she used it only once for irritation. Six days after transplantation, she developed grade 3 mucositis and underwent nutritional management via infusion. From 2 weeks after transplantation, her oral condition improved slightly (Figure 2). At 20 days after transplantation, the mucositis gradually improved, and brushing became possible. At 27 days after transplantation, the mucositis disappeared. The gingival swelling became mild, redness improved, and no gingival bleeding was detected during brushing (Figure 3). She was discharged 84 days after transplantation. Although there was no change in the degree of tooth movement, the gingival pocket depth and gingival swelling in the lower anterior region improved, and the O'Leary PCR value was 24.1%, which was lower than that before transplantation (Table 4).

## 3. Discussion

CHS is characterised by severe periodontitis from childhood due to its adverse effects on immunity; however, its management is rarely reported due to its low incidence. Shibutani et al. [4] conducted long-term follow-up observations in a patient with CHS and reported that periodontal treatment resulted in a limited response. Our patient appeared to have atypical CHS as she had not been diagnosed even until the age of 16 years. Irrespective of age, her periodontitis was moderate, with a periodontal pocket depth of 5–6 mm and tooth mobility. She often experienced fever and salivary gland inflammation, possibly caused by oral bacterial infection.

The treatment for CHS is bone marrow transplantation; its success depends on professional oral health care and self-administered oral care. CHS can be associated with cognitive and neurological impairments [5]. Certain et al. [6] described patients with CHS with low intellectual capacity, mental retardation, and poor school performance. Our patient was borderline in this respect as she has an intelligence quotient of 75. Despite being aged 18 years, she was initially scared of using the ultrasonic scaler and it was difficult for her to brush her teeth independently. The interventions were initiated 7 weeks before bone marrow transplantation, and subsequently, she has become accustomed to dental instruments and dental hygienists. In addition, we provided professional oral health care and self-care guidance to the patient and her parents. Kashiwazaki et al. [7] reported that professional oral health care reduces the incidence of oral mucositis and febrile neutropenia after bone marrow transplantation. Yoneyama et al. [8] reported that control of dental plaque by professional brushing combined with frequent tooth cleaning and self-administered oral care could reduce microbial counts.

In the present case, the chemo-induced oral mucositis progressed to grade 3 and required tube feeding, but there was no febrile neutropenia. Oral mucositis was alleviated using a mouthwash containing lidocaine. Episil® has recently been used to control pain in oral mucositis. It protects the oral mucosa and controls and alleviates oral pain caused by stomatitis associated with chemotherapy and radiotherapy through a physical mode of action. It became commercially available in Japan in 2018. It protects the surfaces of ulcers and erosions and relieves pain by forming a bioadhesive film. Hadjieva et al. [9] reported that the use of Episil® in patients who underwent radiotherapy for head and neck cancer provided significant pain relief.

Because Episil® is a spray, our patient could not use it because it caused painful irritation during spraying. We determined that as the mucositis had progressed to grade 3, the spray should have been used earlier. Eventually, her mucositis and oral hygiene improved, and she was successfully discharged.

This is the first report of the oral management of a patient with CHS undergoing haematopoietic stem cell transplantation to the best of our knowledge. We performed oral care in this patient with CHS accompanied by periodontitis, and it contributed to successful haematopoietic stem cell transplantation and improved her periodontitis. Periodontitis tends to become severe in patients with CHS; however, early management is important as stem cell transplantation is performed for the treatment.

## Figures and Tables

**Table 1:** Periodontal examination chart 7 weeks before the transplantation.

Grade of mobility	—	1	1	1	1	1	1	1	1	1	1	1	1	1	1	—
Probing depth	—	5	5	5	5	5	6	6	6	5	6	5	5	5	5	—
Tooth	8	7	6	5	4	3	2	1	1	2	3	4	5	6	7	8
Tooth	8	7	6	5	4	3	2	1	1	2	3	4	5	6	7	8
Probing depth	—	5	5	5	5	5	5	5	5	5	5	5	5	5	5	—
Grade of mobility	—	1	1	1	1	1	1	1	1	1	1	1	1	1	1	—

Plaque control record (O'Leary) 80%.

**Table 2:** Treatment schedule and oral condition before the transplantation.

Week	−7	−6	−5	−4	−3	−2	−1	0 (transplantation)
Pretransplant treatment							VP-16, BU, CY	
O'Leary PCR (%)	80.0	74.1	64.3	54.5	53.0	58.0		
Stomatitis (grade)							1	
Treatment for stomatitis								

**Table 3:** Treatment schedule and oral condition after the transplantation.

Day	0	1	2	3	4	5	6	7	8	9	13	14	15	16	20	84
Immuno-suppressive therapy			MTX													
O'Leary PCR(%)		2														24.1

Stomatitis(grade)							3				2				1	

Treatment for stomatitis	Mouthwash containing lidcaine					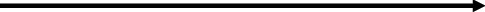										
							episil®									

**Table 4:** Periodontal examination chart 12 weeks after the transplantation.

Grade of mobility	—	1	1	1	1	1	1	1	1	1	1	1	1	1	1	—
Probing depth	—	3	3	3	2	2	3	3	2	2	2	2	3	3	3	—
Tooth	8	7	6	5	4	3	2	1	1	2	3	4	5	6	7	8
Tooth	8	7	6	5	4	3	2	1	1	2	3	4	5	6	7	8
Probing depth	—	3	2	2	2	3	3	2	2	2	2	2	3	4	3	—
Grade of mobility	—	1	1	1	1	1	1	1	1	1	1	1	1	1	1	—

Plaque control record (O'Leary) 24.1%.
